# Association of STAT6 gene polymorphism with atopic asthma among Yemeni children in Sana’a city, Yemen

**DOI:** 10.1186/s12887-025-05710-9

**Published:** 2025-05-15

**Authors:** Haitham Abdulwahab Masood, Arwa Mohammed Othman, Najla Nasr Addin Al-Sonboli, Faiza Abdulnoor Ghlab, Naif Mohammed Al-Haidary, Muhanna M. Al-Shaibani

**Affiliations:** 1 Department of Medical Microbiology and Immunology, Faculty of Medicine and Health Sciences, Sana’a University, Sana’a, Yemen; 2 Department of Pediatrics, Faculty of Medicine and Health Sciences, Sana University, Sana’a, Yemen; 3 Department of Medical Microbiology and Immunology, Faculty of Medicine and Health Sciences, Taiz University, Taiz, Yemen

**Keywords:** rs324011 SNP, IgE, IL-13, Eosinophils, Atopic asthma

## Abstract

**Background:**

Asthma is the most common chronic illness in children and is characterized by airway hyperresponsiveness, increased production of mucus, and significant inflammation. The signal transducer and activator of transcription 6 (*STAT6*) is a crucial gene in immune response, specifically in atopic reactions. It plays a role in the IL-4 and IL-13 signaling pathways in asthma and allergies.

**Objective:**

This study aimed to determine the association between the *STAT6* rs324011 gene polymorphism and atopic asthma among Yemeni children as well as to investigate the impact of the *STAT6* rs324011 polymorphism on IL-13, total IgE, and eosinophils.

**Methods:**

This study included 75 Yemeni children diagnosed with bronchial asthma and 75 healthy controls matched for age and sex. The *STAT6* polymorphism (rs324011) was genotyped using RFLP PCR, the IL-13 serum level was measured via ELISA, and the serum IgE level was measured via electrochemiluminescence.

**Results:**

Under a recessive model, the TT genotype of the *STAT6* polymorphism rs324011 was significantly associated with an increased risk of atopic asthma compared to the CC and CT genotypes (χ^2^ = 6.6, OR = 2.5, CI = 1.2–5, *p* = 0.01). IgE levels among asthmatic children were significantly elevated in individuals with the TT genotype compared with those with the CC or CT genotypes (*p* = 0.04).

**Conclusion:**

The TT genotype and T allele of the *STAT6* rs324011 polymorphism may be associated with increased susceptibility to pediatric asthma among Yemeni children.

## Introduction

Allergic asthma is a respiratory illness characterized by airway inflammation and hyper-responsiveness caused by exposure to airborne allergens. These allergens cause immediate bronchoconstriction, followed by a late-phase inflammatory reaction. Allergic asthma is the most common phenotype of asthma. It accounts for up to 80% of pediatric asthma cases and almost half of adult asthma cases [1].

Asthma can develop at any age; however, most cases occur before the age of 25 years. Despite its complexity, involving many genes and probably gene-environment interactions, asthma demonstrates a heritability of approximately 60%. These findings suggest genetic and environmental factors contribute to pathogenesis [2]. Allergic asthma is related to type I hypersensitivity reactions in which different allergens, such as pollen, and dust mites, induce type 2 immune responses, which are defined by the generation of interleukin-4 (IL-4), IL-5, and IL-13 and class switching to IgE antibodies [3]. These cytokines, IL-4, IL-5, and IL-13 are primarily produced by Th2 cells. However, other immune cells, such as type 2 innate lymphoid cells (ILC2s), mast cells, eosinophils, and basophils, also contribute to their production [4].

STAT6 regulates immunological responses and plays a crucial role in the development of allergic reactions and efficient immunity against helminth parasites [5]. It is a key regulator of Th2-mediated allergic inflammation via the IL-4 JAK/STAT signaling pathway [6]. STAT6 stimulates immune cell responses through IL-4 and IL-13 signaling. This stimulates the proliferation of B and T cells, the development of alternatively activated macrophages, and the modulation of IgE class switching in B cells via the activation of NF-κB [7]. The human *STAT6* gene is located on chromosome 12q13.3-q14.1, spanning a total length of 19 kb and comprising 23 exons crossed with 22 introns. The 5’UTR (untranslated region) includes the first two exons and part of the third exon, as well as a major part of the last exon. STAT6 generates many mRNA isoforms [8]. The STAT6 protein consists of 847 amino acids [9], STAT proteins have six domains: the N-terminal domain (ND), coiled-coil domain (CCD), DNA-binding domain (DBD), linker domain (LD), Src homology 2 (SH2) domain, and transcription activation domain (TAD) [10]. Several polymorphisms within the STAT6 gene have been investigated in relation to allergic disorders. Among these, rs324011, rs324015, and rs4559 have been studied for their influence on IgE levels, cytokine signaling, and Th2-mediated responses [11–13].

We hypothesized that the *STAT6* rs324011 polymorphism is associated with an increased susceptibility to atopic asthma among Yemeni children, with the TT genotype conferring a higher risk. The rs324011 SNP is located in the second intron of the *STAT6* gene, involving a substitution of cytosine (C) with thymine (T) [14]. Our hypothesis aligns with prior evidence from Middle Eastern and South Asian populations. For instance, the TT genotype of rs324011 has been associated with a 2.1-fold increased asthma risk in Saudi Arabian children [11] and a 2.8-fold risk in the Egyptian study [12]. Similarly, studies in Pakistani populations linked this SNP to elevated IgE levels and non-atopic asthma [15]. The consistency of these findings across genetically and environmentally distinct populations underscores the potential universality of STAT6’s role in asthma pathogenesis, particularly in IgE-mediated pathways. We also examined how this genetic variation might influence IL-13, IgE, and eosinophil levels.

### Methodology

This is a case-control study that included 75 Yemeni children diagnosed with atopic asthma who were compared with 75 healthy controls matched for age and sex (Table 1). Asthmatic children were selected from hospitalized patients in the pediatric department of Al-Sabeen Hospital for Women and Children, Al-Thawra General Hospital and a pediatric chest clinic in Sana’a City, with confirmation of asthma diagnosis by a pulmonologist based on the criteria of the Global Initiative for Asthma (GINA) guidelines [16].

**Table 1:** Demographic characteristics of patients and controls

Variable		Case (*n* = 75)		Control (= 75)	
		No.	%	No.	%
Age	1–5 years	23	31%	24	32%
	6–10 years	31	41%	33	44%
	11–14 years	21	28%	18	24%
Gender	Male	40	53%	37	49.3%
	Female	35	47%	38	50.7%
Residence	Urban	62	82.7%	71	94.7%
	Rural	13	17.3%	4	5.3%
Family history	No	29	39%	75	100%
	Yes	46	61%	0	0%
Other allergic disease	No	48	64%	75	100%
	Yes	27	36%	0	0%

All participants in this study were Yemeni. To minimize the possibility of population stratification, participants from other ethnic backgrounds were not included. All participants underwent comprehensive medical assessments, including complete medical history, physical examinations, pulmonary function tests, and laboratory investigations. Children with non-atopic asthma or other respiratory or chronic illnesses were excluded from the study.

### Sample collection

Ten ml peripheral blood samples were obtained from all study participants. Blood (2.5 ml) was collected in two EDTA-containing tubes, and 5 ml was collected in a plain gel tube. EDTA blood samples were used for hematological assays and DNA extraction. The extracted DNA samples were frozen at -20 °C for subsequent genetic analysis. The serum was separated from the gel plain tube after blood clotting and stored for immunological analysis.

### Measurement of serum IgE levels

Total IgE levels were quantified via electrochemiluminescence immunoassay (ECLIA) via a Cobas e 411 analyzer (Roche Diagnostics, Swiss). The assay was carried out according to the manufacturers’ instructions. This assay involves a sandwich immunoassay (Roche Diagnostics, Swiss). The assay was performed by mixing 10 µl of antigen with biotinylated and ruthenium-labeled monoclonal IgE-specific antibodies to form a sandwich complex. Streptavidin-coated microparticles were added to bind the complex to the solid phase. The reaction mixture was transferred to a measuring cell where microparticles were magnetically captured on the electrode surface. Unbound substances were removed via ProCell/ProCell M. Applying a voltage to the electrode triggered chemiluminescence, which was measured via a photomultiplier. The results were obtained through a 2-point calibration method using a calibration curve and master curve from the reagent barcode.

### Measurement of Interleukin 13 (IL-13)

IL-13 levels were measured using enzyme-linked immunosorbent assay (ELISA) performed on plate reader (Meril, India). This assay employed a sandwich ELISA technique with commercially available kit (Elabscience, USA) according to the manufacturer’s protocol. Serum samples were added to a micro-ELISA plate precoated with an antibody specific for IL-13. Biotinylated detection antibody and avidin-HRP conjugate were sequentially added and incubated. Unbound components were washed away, and a substrate mixture was added, resulting in a blue color in the presence of IL-13. The reaction was stopped, causing the color change to yellow. The optical density was measured at 450 nm, with higher values indicating higher levels of human IL-13. The IL-13 concentrations in the samples were determined using a standard curve.

### Measurement of eosinophil count

Ethylene diamine trisodium acetate (EDTA) collected venous blood was assayed on a Sysmex-XT-2000i auto analyzer (Sysmex, Japan). Eosinophil proportions were calculated as a percentage of total white blood cells (WBCs) from complete blood count (CBC) with differential analysis.

### Genetic analysis

DNA was extracted from whole blood using an extraction kit (Promega, USA) according to the manufacturer’s protocol. The genotype of the *STAT6* rs324011 SNP was determined using the PCR-RFLP method. The PCRs (50 µl) included 2 µl of DNA (5–15 µg), 25 µl of DreamTaq Green Master Mix (Thermo Scientific, USA), 1 µl of forward primer (10 pmoles/µl), 1 µl of reverse primer (10 pmoles/µl) (Apical Scientific, Malaysia), and 21 µl of nuclease-free water. The reaction mixture was analyzed using a thermal cycler (Biometra, Germany) (Table 2).

**Table 2:** Primer sequences and PCR protocol

SNP	primer	Sequence	PCR Program
STAT6 rs324011	STAT6-Fwd	5’-CTC TTC CCA CCC CTG TGT CTA TC-3’	1 cycle 95 °C 3 min; 35 cycles 95 °C 30 s, 67 °C 30 s, 72 °C 1 min; 1 cycle 72 °C 5 min
	STAT6-Rev	5’-TCC CAT AGA TAG CCC TCC TAG GTA C-3’	

Following the PCR procedure, the amplicon was digested using the BshNI restriction enzyme (Thermo Scientific, USA). BshNI recognizes and cut the DNA at the sequence 5′-G↓GYRCC-3’ and its complementary 3’-CCRYG↑G-5’, leaving sticky ends with a 5’-G overhang. The resulting fragments were visualized on a 2% agarose gel stained with ethidium bromide and run alongside a 100 bp DNA ladder marker (Kapa Biosystems, Spain). The homozygous CC genotype produces a single band at 132 bp. In contrast, the homozygous TT genotype yields two bands at 25 bp and 107 bp. The heterozygous CT genotype displays three bands at 132 bp, 107 bp, and 25 bp.

### Statistical analysis

Data were analyzed using mean and standard deviation, median, range, absolute and relative frequencies as appropriate. The chi-square test, or Fisher’s exact test, was used. To compare means between groups, one-way ANOVA was applied for normally distributed data, whereas the Kruskal-Wallis H test was used for nonparametric data. The strength and direction of relationships between continuous variables were assessed using Pearson or Spearman correlations, depending on the data distribution. Odds ratios with 95% confidence intervals (CIs) were calculated to assess genotype differences between cases and controls. Statistical significance was defined as a p-value less than 0.05. All analyses were conducted using IBM SPSS Statistics version 22.

## Results

Demographic characteristics of the study population are summarized in Table 1. Most participants were aged 6–10 years (41% cases, 44% controls), with fewer participants aged 11–14 years (28% cases, 24% controls). Most children with asthma lived in urban areas (82.7%) compared to rural areas (17.3%), suggesting a possible environmental influence. 61% of children with asthma had a family history of asthma, and 36% had other allergies, while these factors were absent in the control group, this difference highlights the significant role of family history and allergies in asthma susceptibility. The genotype frequencies of the STAT6-rs324011 polymorphism in the control group were aligned with Hardy‒Weinberg equilibrium expectations (χ² = 2.7, *p* = 0.1) (Table 3). The TT genotype under the recessive model was significantly more common in asthmatic children (44%) than in controls (24%), with a 2.5-fold increased risk of asthma (OR = 2.5, 95% CI = 1.2–5.0, *p* = 0.01) (Table 4). This indicates that Yemeni children with the TT genotype might be 2.5 times more likely to develop asthma compared to those with CC/CT genotypes, with the true population risk ranging from moderate (lower CI = 1.2) to high (upper CI = 5.0). Additionally, the frequency of the T allele was significantly higher in cases (58%) compared to controls (44%), with an odds ratio of 1.8 (OR = 1.8, 95% CI = 1.1–2.8, *p* = 0.021) (Table 5). The T allele is significantly associated with asthma, suggesting that individuals carrying this allele may have an increased risk of developing atopic asthma.

**Table 3:** Frequencies of genotype under Hardy-Weinberg equilibrium

Genotype	CC	CT	TT	χ ^2^	*P*
Observed genotype	27	30	18	2.7	0.1
Expected genotype	23.5	37	14.5		

χ^2^ = Chi-square test, p-value = probability value

**Table 4:** Comparison of STAT6 rs324011 in the dominant/recessive model between atopic asthma children and controls

SNP	Genotype	Model	Case (*n* = 75)	Control (*n* = 75)	OR	CI 95%	χ 2	*P* value
rs324011	CC	Dominant	21 (28%)	27 (36%)	0.7	0.3–1.4	1	0.3
	CT, TT		54 (72%)	48 (64%)	1.4	0.7–2.9		
	CT, CC	Recessive	42 (56%)	57 (76%)	0.4	0.2–0.8	6.6	0.01
	TT		33 (44%)	18 (24%)	2.5	1.2-5		

χ2 = chi-square test, p value = probability value, OR = odds ratio, CI = confidence interval

**Table 5:** Alleles frequency in atopic asthma children and controls

SNP	Genotype/Allele	Case (*n* = 75)	Control (*n* = 75)	OR	CI 95%	χ ^2^	*p* value
rs324011	C allele	63 (42%)	84 (56%)	1.8	1.1–2.8	5.9	0.021
	T allele	87 (58%)	66 (44%)				

χ^2^ = chi-square test, p value = probability value, OR = odds ratio, CI = confidence interval

**Fig. 1:**
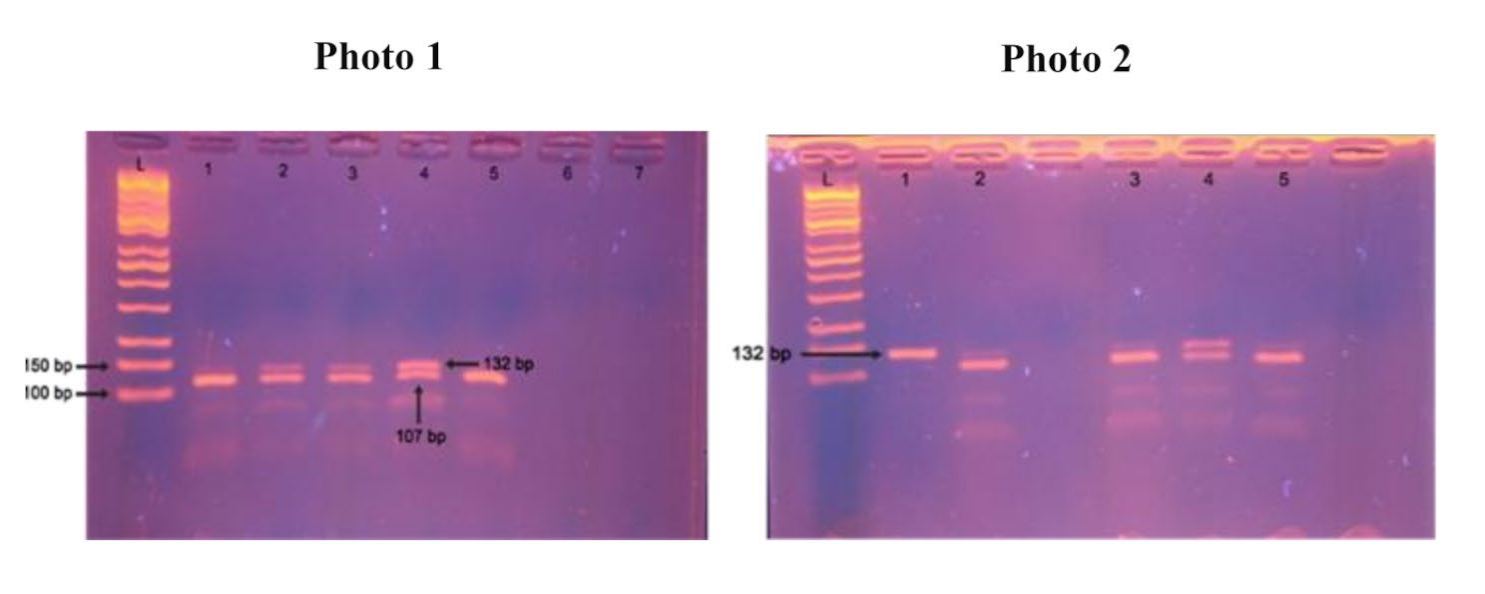
Genotype results. Illustrates the genotyping results for the *STAT6* rs324011 polymorphism using PCR-RFLP. Figure 1** Photo 1** shows the gel electrophoresis results for the STAT6 rs324011 polymorphism. The lanes 2, 3, and 4 are heterozygous (CT) with three bands at 132, 107, & 25 bp. The lanes 1 and 5 were homozygous polymorphic (TT) with two bands at 107 & 25 bp. Figure 1** Photo 2** further validates these patterns, with lanes 2, 3, 4, and 5 were heterozygous (CT) with three bands at 132, 107, and 25 bp. Lane 1 was homozygous wild (CC) with a single band at 132 bp. The 25 bp band appears obscure due to its small molecular size

Children carrying the TT genotype had significantly higher IgE levels (499 ± 211 IU/ml) than those with the CC (387.3 ± 147 IU/ml) or CT (376 ± 163 IU/ml) genotypes (*p* = 0.04). However, no significant differences were observed in IL-13 levels among the genotypes CC (75 pg/ml, range: 49–182), CT (71 pg/ml, range: 44–211), and TT (70 pg/ml, range: 35–180) genotypes (*p* = 0.7). Similarly, eosinophil proportions showed no significant variation among genotypes, with CC (7.1 ± 3.08%), CT (7.8 ± 3.06%), and TT (7.55 ± 3.06%) (*p* = 0.7) (Table 6).

**Table 6:** Association of STAT6 rs324011 genotype with IgE, IL-13, and eosinophil levels in atopic asthmatic children

Test	STAT6 rs324011 genotype			*p* value
	CC (*n* = 21)	CT (*n* = 21)	TT (*n* = 33)	
IgE level (IU/ml)	387.3 ± 147.3	376 ± 163	499 ± 211	0.04*
IL-13 level (pg/ml)	75 (49–182)	71 (44–211)	70 (35–180)	0.7**
Eosinophil %	7.1 ± 3.08	7.8 ± 3.06	7.55 ± 3.06	0.7*

*ANOVA test ** Kruskal‒Wallis’s test

## Discussion

Asthma is the most chronic disease in children and is characterized by airway hyperresponsiveness, excessive mucus production, and pronounced inflammation [17]. Allergic asthma is most prevalent in early childhood and decreases with age, whereas non-allergic asthma remains rare until it peaks in late adulthood. After approximately 40 years of age, most asthma cases are nonallergic [18].

Our findings reveal a significant association between the *STAT6* rs324011 polymorphism and the risk of developing atopic asthma. Specifically, under the recessive inheritance model, the TT genotype was significantly more common among asthmatic children (44%) than among controls (24%) (OR = 2.5, 95% CI = 1.2–5.0, *p* = 0.01). This suggests that Yemeni children with the TT genotype are 2.5 times more likely to develop asthma compared to those with CC/CT genotypes, with the true population risk ranging from moderate (CI = 1.2) to high (CI = 5.0). These results indicate a potential association between the TT genotype and elevated asthma risk. This finding suggests that the *STAT6* rs324011 polymorphism may be a potential candidate marker for identifying individuals at increased risk of asthma, particularly in high-risk families. Furthermore, understanding the role of STAT6 in asthma pathogenesis could lead to the development of targeted therapies. For instance, STAT6-immunomodulatory peptides have been shown to reduce type 2 innate lung inflammation and Th2 adaptive immunity, suggesting potential for reducing asthma symptoms [19]. However, additional research is needed to confirm these findings and to explore the functional consequences of the TT genotype. Our results are consistent with those of a study conducted in Saudi Arabia [11], which reported that the TT genotype was significantly associated with asthma compared with the CC and CT genotypes. However, their study differed from ours in that they reported no statistically significant difference in the frequency of C and T alleles between patients and controls. The difference in sample sizes between the studies may have led to variations in statistical power, potentially explaining the observed discrepancy. Further research exploring gene-environment interactions is needed to investigate this possibility. Furthermore, a study from Egypt [12] revealed that both the rs324011 TT genotype and T alleles were significantly associated with increased susceptibility to developing bronchial asthma. Similarly, a Pakistani study indicated that the rs324011 polymorphism was significantly associated with non-atopic asthma [13]. These studies, in conjunction with our findings, provide further evidence supporting the role of the *STAT6* rs324011 polymorphism in asthma susceptibility across diverse populations and asthma. This consistency strengthens the potential clinical utility of this polymorphism as a risk marker for asthma.

STAT6 is critical in the signaling pathways associated with cytokines IL-4 and IL-13, which play essential roles in the pathogenesis of many allergic disorders [20]. The *STAT6* rs324011 variant may increase asthma risk because STAT6 mediates the biological effects of a cytokine necessary for type 2 differentiation of T cells and B-cell survival, proliferation, and class switching to IgE [21]. Compared with that in healthy controls, the transcriptional activity of mutant STAT6 was increased even without IL-4 stimulation, and the phosphorylation of STAT6 was more strongly induced by IL-4 stimulation. The patient’s lymphoblastoid cell lines showed nuclear presence of STAT6 protein even without IL-4 stimulation [22]. Our study investigated the relationships between three genotypes (CC, CT, and TT) and IgE, IL-13, and eosinophil levels in children with atopic asthma. While individuals with the TT genotype presented significantly higher IgE levels than those with the CC or CT genotypes, no significant differences were found in IL-13 or eosinophil levels. These results partially aligned with those of [12], who reported significant associations between *STAT6* SNP genotypes and IgE and eosinophil levels. The lack of significant differences in IL-13 or eosinophil levels may be attributed to the fact that STAT6 primarily regulates IgE production through the B cell pathway, whereas IL-13 and eosinophil levels are likely influenced by other pathways, such as those involving IL-5 or GM-CSF. Additionally, IL-13 and eosinophil levels may be modulated by independent genetic, epigenetic, or environmental factors, which were not directly related to STAT6.

*STAT6* SNPs can alter gene expression, suggesting that these variants are expression SNPs associated with transcription. Consequently, changes in the mRNA expression levels of the five major IL-4/IL-13 pathway genes, which are DNA variants, lead to gene expression changes, which lead to asthma susceptibility and subsequently elevated serum IgE [23].

Early asthmatic responses are triggered by T cells, derived cytokines, IgE, mast cells, and recruitment and activation of eosinophils, which appear to contribute to the persistent asthma phenotype with chronic airflow obstruction [2]. IgE is responsible for the release of several asthma-associated inflammatory mediators from mast cells, such as histamine and prostaglandins [24]. The functions of specific cytokines in asthma are clear; specifically, IL-13 plays an important role in eosinophil accumulation and is considered a critical factor in IgE synthesis by B cells, differentiation of naïve T cells into Th2 effector cells, AHR, and airway inflammation [2]. Furthermore, IL-13 contributes to regulation and driving type 2 inflammation. It plays a key role in asthma by promoting airway hyper-responsiveness, mucus secretion, and airway remodeling [25]. Eosinophil levels are elevated in both the bloodstream and airways of many asthma patients [26]. The influx of eosinophils and their activation of the bronchial mucosa is a characteristic feature of asthma [27]. Eosinophilic airway inflammation is observed in approximately 40–60% of individuals with severe asthma [28].

Our findings revealed that 82.7% of children with asthma resided in urban areas compared to 17.3% in rural regions, which may reflect higher exposure to environmental pollutants. Environmental factors, particularly air pollution, play a significant role in the higher prevalence of asthma in urban areas. Urban environments typically have higher levels of outdoor air pollutants such as ozone (O₃), nitrogen dioxide (NO₂), sulfur dioxide (SO₂), and particulate matter (PM), which are linked to asthma development and exacerbation. Chronic exposure to traffic-related pollution can impair lung function in children, increasing the risk of asthma. Additionally, indoor air quality in urban settings, influenced by pollutants from building materials, cleaning products, and other sources, further contributes to asthma risk and symptoms in children [29, 30].

This study has some limitations. **First**, while our sample size was determined through rigorous power analysis and sufficient to detect the observed associations, a larger sample size incorporating diverse geographic regions within Yemen would enhance generalizability and reduce selection bias. Such efforts would also better capture population-wide genetic variability. However, limited funding caused by the ongoing conflict in Yemen restricted the ability to include a larger sample size.

**Second**, while this study provides valuable insights into the association between the *STAT6* rs324011 polymorphism and atopic asthma in Yemeni children, it’s important to recognize the limitations of focusing on a single SNP. Analyzing additional SNPs in *STAT6* (e.g., rs324015, rs3024944, rs71802646) and other genes (e.g., *IL1RL1*,* GATA3*,* ADAM33*,* TSLP*,* FOXO3a*) would expand the scope of genetic analysis and provide a comprehensive understanding of asthma susceptibility in the population. Future studies in Yemen should prioritize multi-SNP analyses and gene-environment interplay to refine predictive models and therapeutic targets for this complex disease.

**Third**, our study did not include environmental factors such as air pollution, allergens, dietary factors, or socioeconomic conditions. Future research in Yemen should incorporate gene-environment interaction analyses to evaluate how these external factors influence the genetic risks associated with *STAT6* variants.

**Finally**, while our current study focused on validating the genetic association between rs324011 and asthma susceptibility, detailed clinical data regarding treatment failure, acute exacerbations, or advanced therapies were not comprehensively captured. Future studies should integrate clinical parameters with genetic markers would significantly enhance the translational relevance of these findings.

## Conclusions

Children with the TT genotype or the T allele of the *STAT6* rs324011 polymorphism might be at a greater risk of developing asthma than children without this genetic variation. This finding indicates a possible association between this genetic variation and the development of asthma in Yemeni children. Further research is necessary to investigate additional genetic markers and their interactions with environmental factors.

## Data Availability

The data that support the findings of this study are available for interested researchers upon reasonable request from the corresponding author.

## Declarations

### Clinical trial number

Clinical trial number: not applicable.

## Abbreviations

STAT6: Signal transducer and activator of transcription 6
ND: N-terminal domain
CCD: Coiled-coil domain
DBD: DNA-binding domain
LD: Linker domain
SH2: Src homology 2
TAD: Transcription activation domain
GINA: Global Initiative for Asthma
Cis: Confidence intervals
OR: Odds ratio
p-value: Probability value
